# Lesions of *Mycobacterium avium* spp. *hominissuis* Infection Resembling *M. bovis* Lesions in a Wild Mule Deer, Canada^1^

**DOI:** 10.3201/eid2607.200187

**Published:** 2020-07

**Authors:** Kirsten M.F. Frayne, Brock R. Chappell, Jennifer L. Davies, Bryan J. Macbeth, Musangu Ngeleka, Jamie L. Rothenburger

**Affiliations:** University of Calgary Faculty of Veterinary Medicine, Calgary, Alberta, Canada (K.M.F. Frayne, B.R. Chappell, J.L. Davies, J.L. Rothenburger);; Banff National Park, Parks Canada Agency, Banff, Alberta, Canada (B.J. Macbeth);; Prairie Diagnostic Services, Saskatoon, Saskatchewan, Canada (M. Ngeleka);; Canadian Wildlife Health Cooperative Alberta Region, Calgary (J.L. Rothenburger)

**Keywords:** Bacteria, bovine tuberculosis, cervids, environment, granuloma, lungs, lymph nodes, mule deer, *Mycobacterium avium*, *Mycobacterium bovis*, *Odocoileus hemionus*, tuberculosis and other mycobacteria, wild animals, zoonotic diseases

## Abstract

We used molecular analyses to confirm *Mycobacterium avium* spp. *hominissuis* infection in lung granulomas and pyogranulomas in the tracheobronchial lymph node in a wild mule deer in Banff, Canada. These lesions are similar to those found in *M. bovis*–infected animals, emphasizing the critical need for disease surveillance in wildlife populations.

In November 2018, a wild yearling male mule deer (*Odocoileus hemionus*) was found dead in Banff National Park, Alberta, Canada. The carcass was submitted to the Canadian Wildlife Health Cooperative Alberta Region at the University of Calgary (Calgary, Alberta, Canada) for diagnostic investigation. The University of Calgary Veterinary Sciences Animal Care Committee approved this research (AC17-0010).

Necropsy revealed that the deer had died of blunt-force trauma, presumably having been struck by a vehicle. During the necropsy, we found mineralized granulomas in the right caudal lung lobe and multifocal pyogranulomatous lymphadenitis in the tracheobronchial lymph node (Figure). We fixed lesion samples in 10% neutral buffered formalin for 48 h, then processed the samples by routine methods; we stained 4-μm-thick sections of paraffin-embedded tissues with hematoxylin and eosin before examination with light microscopy by an anatomic veterinary pathologist (J.L.R.), certified by the American College of Veterinary Pathologists. 

**Figure:**
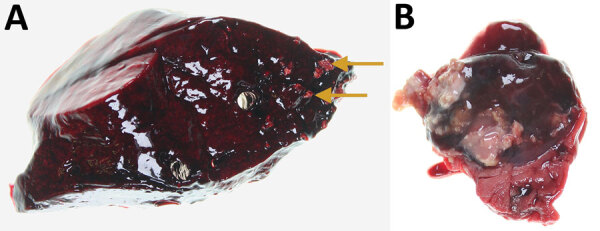
Lung and tracheobronchial lymph nodes from a wild mule deer (*Odocoileus hemionus*) infected with *Mycobacterium avium* spp. *hominissuis*, Banff National Park, Alberta, Canada. A) Mineralized granulomas in the right caudal lung lobe (arrows). B) Pyogranulomatous lymphadenitis of a tracheobronchial lymph node. The lesions resemble those caused by infection with *M. bovis*.

Histopathology of the lung confirmed granulomatous pneumonia. Affected multifocal areas were characterized by central necrotic debris that was variably mineralized, surrounded by macrophage aggregates, multinucleated giant cells, lymphocytes, plasma cells, and variably thick fibrous capsules. Histopathology of the lymph node revealed similar multifocal areas of necrosis, variable mineralization, and infiltration of macrophages in the edges of some affected areas. Because of autolysis and freeze-thaw artifact, further characterization of the inflammatory cell population was not possible. 

We designated the lymph node lesions as pyogranulomatous lymphadenitis because of the suppurative gross appearance of the lymph node combined with the microscopic presence of macrophages and mineralization revealed during histologic examination. We identified acid-fast organisms in multifocal macrophages at the margin of necrotic areas in both the lung and lymph node lesions. We submitted frozen samples to the Prairie Diagnostic Services laboratory (Saskatoon, Saskatchewan, Canada), for further analyses. Results of routine bacterial culture and PCR for *Mycobacterium bovis* were both negative. Subsequently, results were positive from a generic *Mycobacterium* spp. PCR of the lung and lymph node using nested internal primers of 439bp, as described elsewhere (*1*). Genomic sequencing analysis identified *M. avium* spp. *hominissuis* (GenBank accession no. MT012364). 

*M. avium* complex disease, caused by 4 species of *M. avium*, including *M. avium* spp. *hominissuis*, is considered a potentially zoonotic disease of global importance (*4*). *M. avium* spp. *hominissuis* is a rare nontuberculosis mycobacterium that infects a wide range of species, most commonly humans and pigs (*2*,*3*). An opportunistic environmental pathogen that persists in soil and water, *M. avium* spp. *hominissuis* typically infects the lungs and intestinal tract, presumably by inhalation and ingestion, respectively (*4*,*5*). 

In humans, *M. avium* spp. *hominissuis* typically results in chronic granulomatous infections in the lungs and cervical lymph nodes (*6*), most commonly in immunocompromised persons (*3*). In children, infection causes lymphadenitis of the head and neck region (*3*). In pigs and other animals, however, *M. avium* spp. *hominissuis* infection primarily manifests as gastrointestinal disease, with granulomatous lesions in mesenteric lymph nodes and abdominal organs, such as the liver, spleen, and small intestines (*6*). Yoshida et al. described lung granulomas as an incidental finding in a slaughtered steer that were similar to the ones identified in the mule deer in this study (*7*). However, these lesions are an unusual manifestation for this bacterium in animals. More typically, respiratory *Mycobacterium* infections in cattle are caused by *M. bovis,* the causative agent of bovine tuberculosis and a zoonotic pathogen of global importance with extensive regulatory and trade implications (*8*). 

The lesions in this case were morphologically similar to those described in animals infected with *M. bovis*. Pyogranulomas and granulomas in the lungs and cervical lymph nodes are found in wildlife reservoirs of *M. bovis,* including cervids (*8*). The similarity of these lesions emphasizes the importance of continuing surveillance and thorough investigation of suspected *M. bovis* cases. Cattle in Canada are regarded as free of *M. bovis*; however, 3 separate cattle herds in western Canada have tested positive from 2011 through March 2020 (*9*). 

*M. bovis* is endemic in wild wood bison (*Bison bison athabascae*) and elk (*Cervus canadensis*) in 2 other national parks in Canada geographically separated from Banff by hundreds of kilometers (*10*). The presence of wildlife reservoirs elsewhere, combined with the sporadic identification of *M. bovis*–infected cattle herds in Canada, has led to concerns over surveillance in wildlife (*8*,*9*). This case demonstrates that routine disease surveillance activities in wildlife populations, including molecular investigations, are crucial to providing ongoing assurance to agricultural and public health sectors of the absence of *M. bovis* in wild cervid populations outside of known endemic areas.
